# Mozambican *Coffea* accessions from Ibo and Quirimba Islands: identification and geographical distribution

**DOI:** 10.1093/aobpla/plae004

**Published:** 2024-02-06

**Authors:** Luciano Navarini, Davide Scaglione, Lorenzo Del Terra, Simone Scalabrin, Lopes Mavuque, Luca Turello, Rafael Nguenha, Gianluca Luongo

**Affiliations:** illycaffè SpA, via Flavia 110, Trieste, Italy; IGA Technology Services, via Jacopo Linussio 51, Udine, Italy; illycaffè SpA, via Flavia 110, Trieste, Italy; IGA Technology Services, via Jacopo Linussio 51, Udine, Italy; Faculty of Agricultural Sciences, Lúrio University, Campus de Unango, EN733 Km42, Unango, Niassa, Mozambique; illycaffè SpA, via Flavia 110, Trieste, Italy; UNIDO (IET/AGR/RAP), Wagramer Str. 5, 1220 Wien, Austria; UNIDO (IET/AGR/RAP), Wagramer Str. 5, 1220 Wien, Austria

**Keywords:** *Coffea racemose*, *Coffea zanguebariae*, genetic characterization, genetic diversity, Ibo coffee, Mozambique, phylogenetic tree, taxonomy

## Abstract

Mozambique does not have a tradition of farming *Coffea arabica* or *Coffea canephora,* the two species that dominate the worldwide coffee market. However, native coffee plants have been growing spontaneously and in some cases cultivated in the Ibo and Quirimba islands in the north of the country and Inhambane province in the south. Historically there has been confusion over the precise taxonomic classification of these indigenous coffee plants, with different botanists identifying the species as *C. racemosa, C. zanguebariae* or various synonyms of both. The present research aims to clarify the subject and provide new information on these little-described coffee species which may prove valuable as new breeding material for future cultivars, something that is sorely needed to face the present and future challenges of coffee production. Leaf samples were collected from 40 accessions from Ibo Island, Quirimba Island and Inhambane province. The samples were sequenced by whole-genome technology and WGS reads were filtered to identify relevant SNP variants. Diversity among the samples was assessed by PCA, and a phylogenetic tree including several *Coffea* species was built using additional data available in public databases. Experimental data confirm the presence of *C. zanguebariae* as the only coffee species present in both Ibo and Quirimba Islands, while it appears that *C. racemosa* is exclusive to the southern Inhambane province. The present research provides the most detailed analysis so far on the genetic identity of the traditional Mozambican coffee crops. This is the prerequisite for undertaking further scientific studies on these almost unknown coffee species and for starting agronomic development programs for the economic revival of Ibo and Quirimba islands based on coffee cultivation. Furthermore, these species could provide much-needed genetic material for the breeding of new hybrids with the two main commercial coffee species.

## Teaser

Although Mozambique does not have a tradition of farming *Coffea arabica* or *Coffea canephora* (the two species that dominate the worldwide coffee market), native coffee plants are cultivated in the country. Historically there has been confusion over the precise taxonomic classification of these indigenous coffee plants, with different botanists identifying the species as *C. racemosa* and/or *C. zanguebariae* . The present research clarifies the subject by combining direct sampling in the field with WGS genotyping, confirming the presence of *C. zanguebariae* as the only coffee species present in the northern Ibo and Quirimba Islands, while *C. racemosa* is exclusive to the southern Inhambane province. These results provide the most detailed analysis so far of the genetic identity of the traditional Mozambican coffee crops, which may prove valuable as new breeding material to face the present and future challenges of coffee production.

## Introduction

Cabo Delgado, the province comprising the extreme north-eastern region of Mozambique, includes the Quirimbas National Park, established in 2002. Among the several islands that make up the marine element of the park Ibo, Matemo, Quisiva, Quilalea, Quirambo and Quirimba have a long tradition of permanent human occupation. Stretching from Pemba city to the Tanzanian border, the Quirimbas archipelago is a chain of almost 400 km of coral reef. Among the inhabited islands, Ibo Island has been known for almost two centuries as the area where a peculiar type of coffee is grown, called Café do Ibo (Ibo coffee). Ibo coffee does not belong to any of the main commercial coffee species, which are part of the *Coffea* genus that comprises around 130 species distributed through equatorial and sub-equatorial Africa, Madagascar, India, SE Asia and Australasia (Davis and Rakotonasolo 2021). The vast majority of the coffee trade is based on only two species of *Coffea*, namely *C. arabica* L. and *C. canephora* Pierre ex Froehner (Illy and Viani, 2004). *Coffea arabica*, the most important coffee grown commercially, is a tetraploid species native from present-day Ethiopia, characterized by a genetic composition of 44 chromosomes and an approximate genome size of 1.3 Gb. It is however the lone exception in its genus, because all other known *Coffea* species are diploids with 22 chromosomes and an average genome size around 700 Mb (Farah, 2019). Among these diploid species are *C. canephora,* the second most important commercial coffee, *C. liberica* which is also grown commercially to a limited extent, and among others Ibo coffee, which is still grown nowadays in small domestic farms in Mozambique and is unique for being the only coffee grown at sea level in coastal areas.

Ibo coffee was distinguished with the Gold Medal Diploma in Lisbon in 1906, due to its unique characteristics in terms of flavour and aroma (Vasconcellos, 1906:104). The exact identity of Ibo coffee has been the subject of ongoing confusion, due to the presence of at least two native *Coffea* species growing in the coastal areas of Mozambique. In fact, in the Inhambane province of Southern Mozambique another coffee has been traditionally grown called Inhambane coffee, which shares similar morphological characteristics with Ibo coffee ranging from leaves to flowers, fruits and seeds. Thus, Ibo and Inhambane coffees have been routinely confused (Cheney, 1925; Chevalier, 1947; Cramer, 1957; Haarer, 1962; Guerreiro Filho, 1992).

Already in 1897, Froehner had underlined the similarity of Ibo coffee to *C. zanguebariae,* described by Loureiro in 1790 based on material collected from Zanzibar and for this reason named ‘zanguebariae’ (Loureiro, 1790). Over the years, several synonyms were assigned to this species, including but not limited to: *C. ibo* (Froehner, 1897), *C. schumanniana* (Busse, 1902) and *C. zanzibarensis* (Grey, 1927).

*Coffea zanguebariae* is indigenous to southern Tanzania, northern Zimbabwe and northern Mozambique including Ibo and Quirimba islands, occurring in dry deciduous forest and riverine and coastal thicket, at an elevation of 10–350 m. Instead, according to recent studies (Bridson 1982, 1988, 2003; O’Sullivan and Davis 2017; O’Sullivan *et al.* 2017; Davis *et al.* 2021) the coffee indigenous to central and southern Mozambique including the Inhambane province is *C. racemosa*. This species is also present in northern South Africa (Kwa Zulu Natal) and eastern Zimbabwe as a plant of coastal forest, riverine forest, deciduous woodland and bushland at elevations of 0–500m.

*Coffea racemosa* and *C. zanguebariae* both grow in coastal areas and from the phenotypic perspective, *C. racemosa* has slightly smaller and rounder fruits and seeds compared to *C. zanguebariae* (Davis *et al.* 2021). Interestingly, some plants of Ibo coffee (supposedly *C. zanguebariae*) have a rounded fruit similar to that of *C. racemosa*, and this may have further contributed to the confusion between these coffee species. Differently from *C. racemosa*, *C. zanguebariae* has not been the subject of detailed studies and very little is known about its agronomy, chemistry, and sensory properties. A preliminary chemical characterization of samples of Ibo coffee (Abdurabe, 2022) showed a caffeine content of approximately 1% with a variance of about ± 0.3%, which is similar to the caffeine content of 1.06% detected in *C. racemosa* by Anthony *et al.* (1993) and in line with the amount of caffeine present in the seeds of other *Coffea* species (Campa *et al.*, 2005).

**Figure 1.**
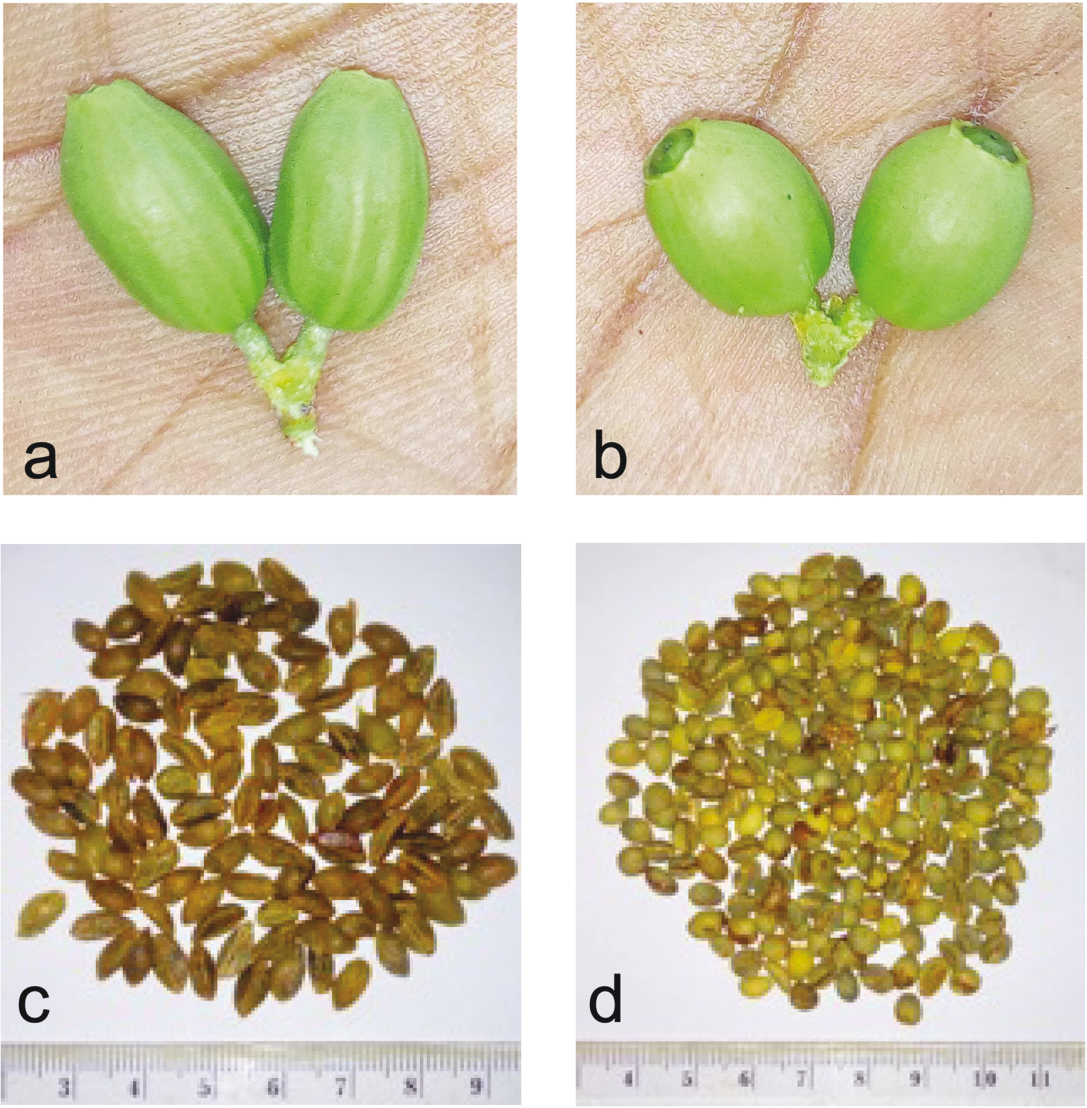
Morphological diversity of Ibo Coffee unripe fruits and seeds. Panel a and c: Variant A fruits and seeds, respectively. Panel b and d: Variant B fruits and seeds, respectively. Adapted from Mavuque (2021a, b).

In a more recent study, about 100 accessions of *Coffea* and *Psilanthus* species were analysed by means of SSR markers, evidencing several phylogenetic lineages consistent with the geographical and ecological distribution of the species (Maurin *et al.* 2007). Polymorphic markers were also used by Davis *et al*. (2021) to assess molecular variation between the closely related *C. racemosa* and *C. zanguebariae*. However, to discriminate the two species the aforementioned study used a small set of only 4 polymorphic markers, and it was limited to a very restricted number (two samples) of *C. zanguebariae* accessions from Ibo Island and no samples from Quirimba Island. In order to provide further evidence in favour of the botanical identity of Ibo coffee, to extend to Quirimba coffee the proper taxonomy and to investigate the distribution of coffee species within these two Mozambican islands, a sampling program has been developed by some of the authors of the present work (Mavuque 2021a, b). In the framework of this program, a preliminary phenotypic characterization study led to the identification of two morphological coffee variants present in both Ibo and Quirimba islands, designated as ‘variant A’ and ‘variant B’ (Abdurabe 2022; Rui Pita 2022). The variants show some differences in habitus, with A presenting scattered branching and fruits at the ends of the branches while B has dense branching and fruiting along the length of the branches (Supplementary Information—Table S1]. The most striking difference however is the size and shape of unripe fruits and seeds. Variant A has bigger fruits with a long elliptical shape and marked longitudinal veins, while Variant B has smaller round fruits with no remarkable veins. The seeds are similarly different, with ‘A’ seeds being flat, pointed and long while ‘B’ seeds are small and rounded (Fig. 1).

This preliminary investigation could not attribute the plants to a precise taxon, hence the use of the generic definition ‘variant’ instead of more proper taxonomic terms like ‘species’ or ‘cultivar’. However, the findings were promising enough to stimulate further investigations aimed at clarifying the botanical classification of these Mozambican coffee plants. This led to the present study which greatly expanded the sample size compared to previous literature, analysing 40 accessions: nine variant A and nine variant B from Ibo Island, nine variant A and nine variant B from Quirimba Island and four *C. racemosa* samples from Inhambane province as control. These accessions were characterized by DNA sequencing and compared to the previous findings by Davis *et al* (2021), resulting in the most accurate analysis of Ibo coffee to this day.

## Materials and Methods

### Study site and experimental design

The study was carried out in the Ibo District (Ibo and Quirimba Islands). In the Ibo District, the average monthly precipitations present a relevant seasonal variation. The wet season lasts between November and April, when there is an amount of precipitations equivalent to about 85% of the total annual value, with March being the wettest month with an average monthly precipitation of about 210 mm. The dry season goes from May to October with monthly average rainfall of less than 50 mm. The average annual temperature is 26.1 °C, with December the hottest month (26.8 °C) and July the coldest (14.8 °C). In the wind system there are three periods with different characteristics: in January and February there are dominant winds from the Northeast and North, from March to August the winds are predominantly from the South and Southeast and between September and December the winds are predominantly from the East and Northeast. In the Ibo District, two types of soils predominate, namely, sandy soils and marine-estuarine sediments. Sandy soils are not very fertile soils for agriculture (apart from planting coconut trees). The natural vegetation cover of these soils is predominantly coastal shrubland. Marine-estuarine sediment soils are clayey soils that support large expanses of mangroves (MICOA, 2012).

**Figure 2.**
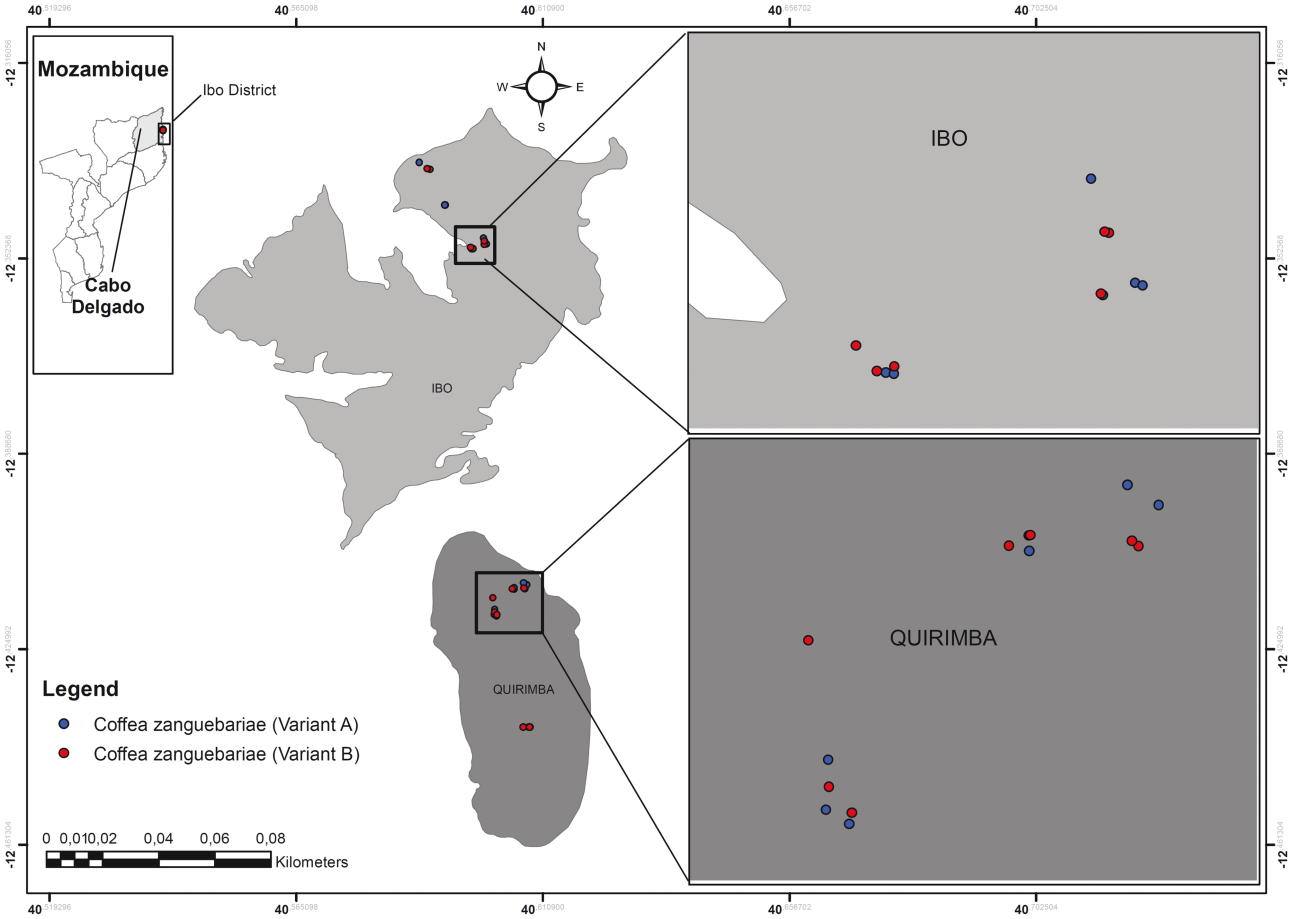
Localization of the sampling sites with position of each sampled accession in Ibo and Quirimba islands (Cabo Delgado Province, Mozambique).

At the sampling sites, coffee plants were randomly selected from domestic gardens and small coffee plantations associated with other crops. In both cases the family-run management is performed in rainfed without regular organic fertilization and proper pruning, giving to the cultivations the connotates of a spontaneous growth. Attention was focussed on selecting an equal number of plants for each of the previously identified variants A and B. The selected coffee plants were georeferenced (Fig. 2) (Supplementary Information—Table S2) to ensure the correct localization of the plants for future observations and investigations.

### Material collection and sample preparation

Young undamaged leaves were collected in Ibo and Quirimba islands from 36 healthy and reproductive plants (18 each from variants A and B) which were appropriately labelled and geolocalized for future reference. Additionally, four different samples of coffee leaves from Inhambane Province (southern districts) were sampled as a control (namely, 1-Maxixi, 3-Inharrime, 9-Zavala, 10-Zavala), presumably belonging to the *C. racemosa* species. Collected leaves were placed in zip-locks, and 10 g of silica gel was added for desiccation and to preserve the leaves’ DNA. Dried leaves were sent to Italy by courier, to keep as short as possible the time between material collection and DNA extraction.

Genomic DNA was extracted from samples by using the Mag-Bind^®^ Plant DNA DS Kit (Omega Bio-Tek Inc, Norcross, Georgia, USA) according to the manufacturer’s instructions with minor modifications. Before processing, 100 mg of samples were grinded by Tissue Lyser II (Qiagen NV, Hilden, Germany) 2 × 30 Hz for 30 s. Subsequent steps were performed according to manufacturer’s recommendations.

### Library preparation and DNA sequencing

Celero™ DNA-Seq Library Preparation Kit (Tecan, Männedorf, Switzerland) has been used for library preparation. Both input and final libraries were quantified by Qubit 2.0 Fluorometer (Invitrogen, Carlsbad, CA, USA) and quality tested by Agilent 2100 Bioanalyzer High Sensitivity DNA assay (Agilent Technologies, Santa Clara, CA, USA). The library was then prepared for whole genome sequencing and sequenced on NovaSeq6000 (Illumina, San Diego, California, USA) in 150 bp paired-end mode.

### Whole-genome SNP diversity

WGS reads available at NCBI SRA (NCBI National Library of Medicine, USA) were retrieved for *C. eugenioides* from accession SRR10513216, for *C. canephora* from SRR8194309, SRR8194229, SRR8194227 and SRR8194312, and for *C. arabica* from SRR9822011, SRR9822012, SRR9822013, SRR9822014 and SRR9822015.

Raw data produced by the Illumina sequencer were processed for both format conversion and de-multiplexing with Illumina Bcl2Fastq v2.20. Adapter sequences were masked with Cutadapt v1.18 (Martin, 2011). Low quality and adapter masked bases were trimmed using ERNE v2.1.1 (Del Fabbro *et al.* 2013). K-mer analysis was carried out with jellyfish v2.3.0 (Marcais and Kingsford, 2011), jellyfish count was used to compute 21-mers, while jellyfish histo was used to extract 21-mers histogram, from which genome size was inferred as in Li *et al*. (2010). A contamination check was carried out with blastn (Altschul *et al*. 1990) versus the NCBI nt database (NCBI National Library of Medicine, USA) and internally developed Perl scripts (Supplementary information - Code 1 and Code 2). Reads were mapped to the *C. canephora* genome sequence (Denoeud *et al.* 2014) with BWA-MEM v0.7.17-r1188 (Li and Durbin, 2009). Aligned sequences considered for downstream analysis were only those mapping to unique positions (mapq >= 10), with PCR-duplicated sequences marked by picard tools (http://broadinstitute.github.io/picard/). Freebayes v2 (Garrison and Marth, 2012) was used for the identification of SNPs and small indels using the following parameters: --min-alternate-count 2 --min-alternate-fraction 0.2 -p 2 --use-best-n-alleles 2. Normalization and filtering of variants was accomplished with bcftools (Danecek *et al*. 2021). Bcftools filter was applied with ‘(FORMAT/DP>=5) --set-GTs’., followed by bcftools view -i ‘(N_PASS(FORMAT/DP>=5)>=10)’ and bcftools view -i ‘(N_PASS(FORMAT/GT==‘RA’) + N_PASS(FORMAT/GT==’AA’)>=1)’. Bedtools (Quinlan and Hall, 2010) subtract and bedtools intersect were used to filter out variants falling within *C. canephora* repetitive sequences and to include only those falling within coding sequences of the *C. canephora* annotated genome. The ‘SNPrelate’ R package (Zheng *et al.*, 2012) was used to execute the PCA analysis with standard parameters, ggplot2 (Wickham, 2016) was used to plot the graphs.

### Phylogenetic tree analysis

The Illumina whole-genome data generated for the 40 samples from this study along with the public available data on NCBI for *C. arabica*, *C. canephora*, and *C. eugenioides* were used to build consensus sequences for the accD-psaI, rpl16, trnL-trnF and ITS loci, as previously used by Davis *et al*. (2021) for discriminating *C. racemosa* from *C. zanguebariae*. Short reads were extracted with bbduk (https://sourceforge.net/projects/bbmap/) with the following parameters: editdistance = 1, minkmerhits = 1, k = 27, using consensus loci as a reference. Such reads were later aligned to the four sequences with BWA-MEM v0.7.17-r1188 (Li and Durbin, 2009). From each alignment, variants were computed with pilon (Walker *et al*. 2014) and a consensus sequence was created with bcftools consensus (Danecek *et al*. 2021). For each sample, the four consensus sequences were concatenated after aggregation with previously sequenced Sanger sequences of *C. costatifructa*, *C. sessiliflora*, four *C. racemosa*, and six *C. zanguebariae* (Davis *et al.* 2021). A multiple alignment of all samples was generated with MAFFT v7 (Katoh *et al.*, 2013). A phylogenetic tree was computed with IQ-TREE (Minh *et al.*, 2020) with substitution model GTR2 and 10000 bootstrap alignments. Graphical representation of the tree was done with FigTree (http://tree.bio.ed.ac.uk/software/figtree/).

## Results

Forty samples of coffee leaves, 18 from Ibo and 18 from Quirimba islands (originating from the Cabo Delgado province in the northern part of Mozambique), one from Inharrime, one from Maxixe, and two from Zavala districts (originating from the Inhambane province in the southern part of Mozambique), were whole-genome sequenced with Illumina technology producing from a minimum of 137 to a maximum of 310 million reads per sample (Supplementary Information—Table S3]. K-mer analysis in eight randomly selected samples estimated the genome size to range from a minimum of 599 Mb to a maximum of 818 Mb, with an average value of 710 Mb, in line with an expected genome size of about 700Mb which is the typical dimension of diploid *Coffea spp.* genomes. Considering a haploid genome size of 700 Mb, the sequencing yield corresponds to a minimum of 29x to a maximum of 66x genome coverage.

A check for possible contaminations was run on 50 000 reads per sample. This revealed that for each sample 75–80% of the reads aligned to some *Coffea* sequences. Minor possible contaminations (including *Homo sapiens* and mildew, e.g. *Lasiodiplodia*) were represented with 1% or less of the reads. Among *Coffea* sequences, 10–30% represented either chloroplastic or mitochondrial sequences.

Due to the lack of a proper reference genome for this *Coffea spp.* the three publicly available diploid genomes of *C. canephora, C. eugenioides* and *C. humblotiana* were considered as reference sequences to which to align the reads, with *C. canephora* selected as a proxy reference genome for reads alignment. After aligning the reads and discarding those mapping to multiple locations in the reference genome, a minimum of 35.62% to a maximum of 48.66% (average 44.50%) of reads were successfully mapped to the *C. canephora* genome. The low mapping rate was expected given the intrinsic phylogenetic distance between the samples and *C. canephora*. To make the study more comprehensive we enlarged the dataset of samples adding WGS reads publicly available at NCBI for *C. eugenioides*, *C. canephora* and *C. arabica*.

**Figure 3.**
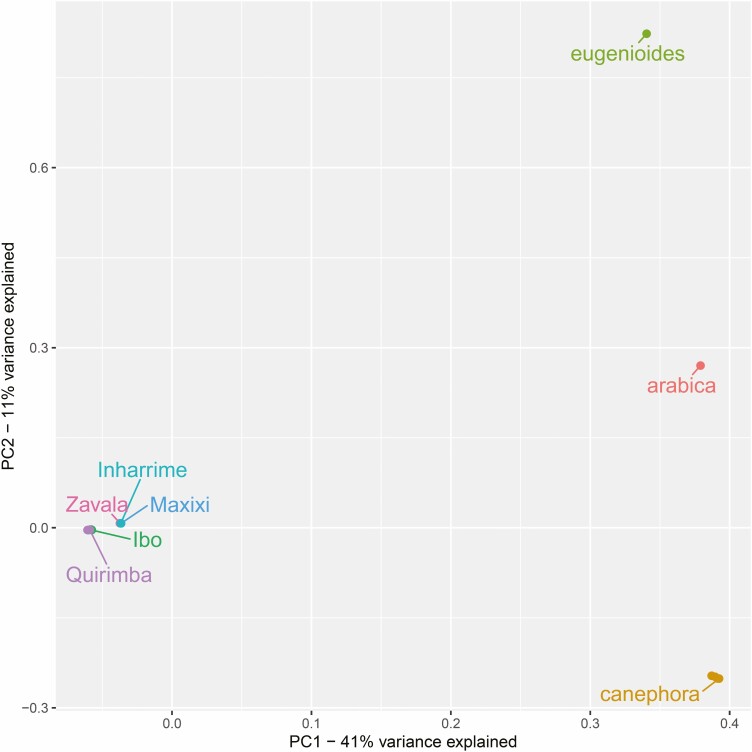
Plot of PC1 and PC2, considering the full dataset including Mozambican samples together with *C. arabica, C. canephora* and *C. eugenioides*. All Ibo samples and all Quirimba samples are plotted as single dots as they do overlap.

Genomic variants were filtered for low-coverage sites and overlaps with *C. canephora* annotated repetitive sequences, retaining only SNPs in coding sequences. A final set of 1 473 750 variants was used in downstream analyses. The PCA analysis showed, as expected, that *C. arabica*, *C. canephora* and *C. eugenioides* samples are genetically distant from Mozambican samples (Fig. 3), that conversely clustered close to each other, likely indicating a very isolated and narrow genetic background. The first component alone of the PCA explained 41% of the variance within the dataset, clearly separating the Mozambican samples from *C. canephora*. PC2 accounted for an additional 11% of the total variance, mostly explained by the divergence between the Mozambican-canephora cluster and *C. arabica* and its maternal progenitor *C. eugenioides* towards the bottom of the graph.

**Figure 4.**
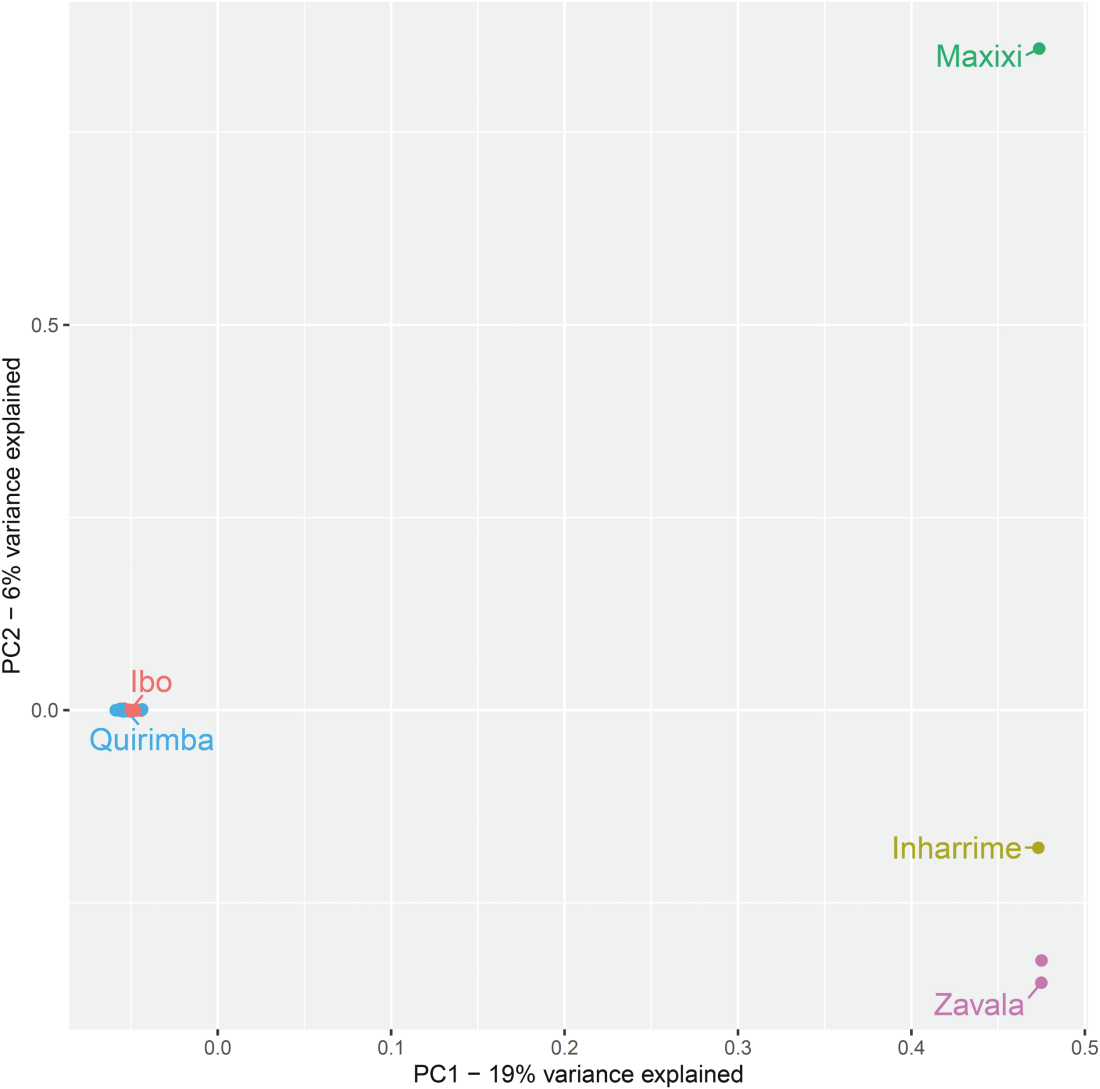
Plot of PC1 and PC2, considering only 40 Mozambican samples from Ibo and Quirimba islands and from the three southern districts. Samples are coloured by provenance, either the two islands or the three southern Mozambican districts.

After excluding *C. arabica*, *C. canephora* and *C. eugenioides*, the PCA provided a much clearer separation of the samples from Southern districts with respect to Ibo and Quirimba samples (Fig. 4). In this case, the first and second components explained respectively 19% and 6% of the variance within the dataset. Finally, considering only samples from Ibo and Quirimba islands, the PCA plot shows a uniform distribution of samples, with Ibo and Quirimba being slightly separated by means of the second component (Supplementary Information—Fig. S1]. Also, the first four components combined only explained 17% of the variance within the dataset. This indicates that the samples very likely derive from the same species, when compared to the amount of variability identified between *C. canephora*, *C. eugenioides* and Inharrime, Maxixi and Zavala samples.

**Figure 5.**
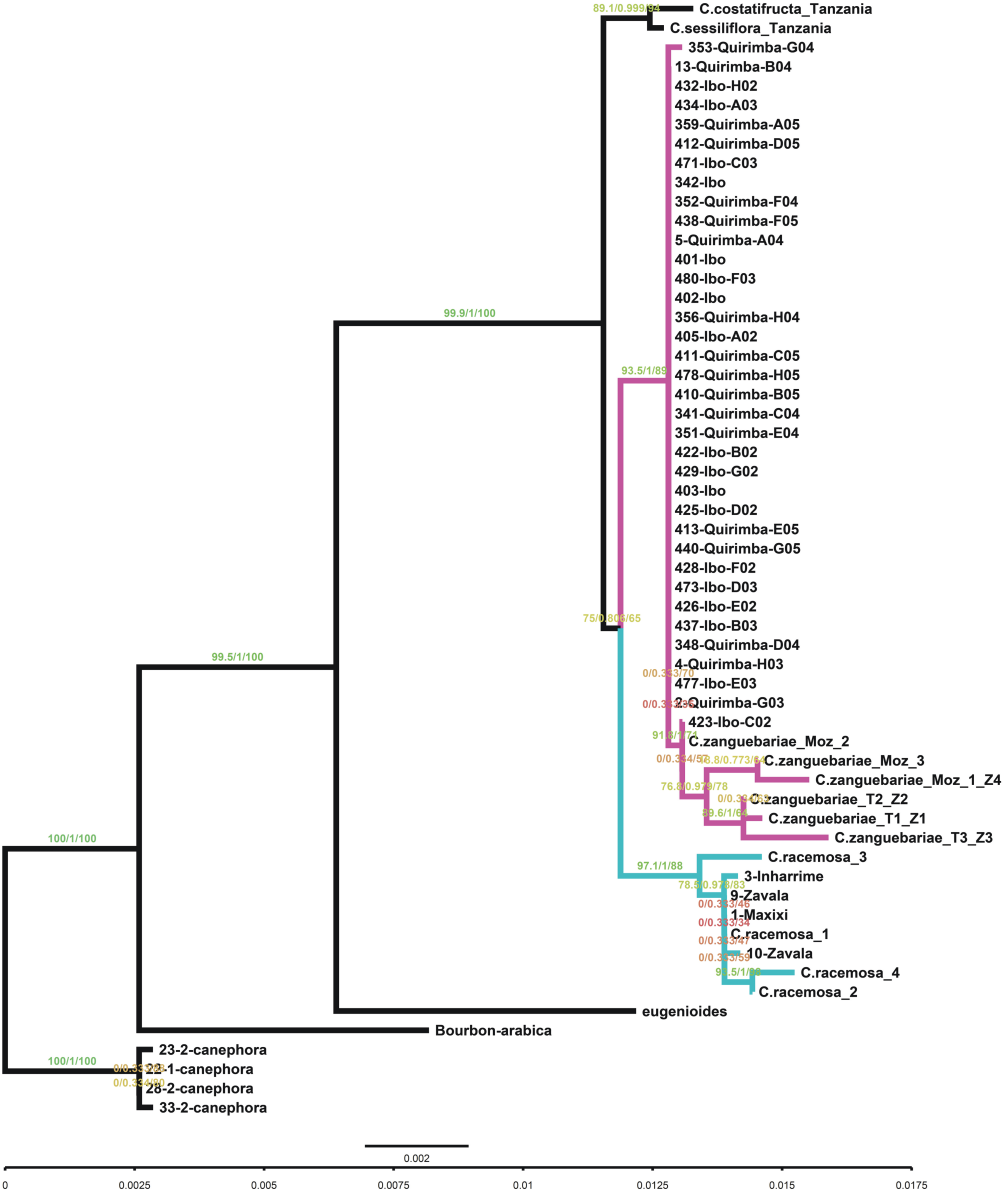
Phylogenetic tree by multi-alignment of three chloroplastic loci and one nuclear locus. Purple branch highlights the Ibo/Quirimba cluster whilst the green branch highlights the *C. racemosa* cluster. Values on each node report SH-aLRT support (%), aBayes support and ultrafast bootstrap support (%) respectively.

To discern whether Mozambican samples are from the same or different species, i.e. *C. racemosa* and *C. zanguebariae*, we computed a phylogenetic analysis based on the genetic diversity detected using the polymorphic markers from Davis *et al.* (2021), comprising three chloroplast loci and one nuclear locus. The phylogenetic tree (Fig. 5) revealed that samples from Ibo and Quirimba islands clustered with *C. zanguebariae* while samples from the southern districts of Mozambique are associated to *C. racemosa* samples, with a 100% posterior probability for both and an 88% and 89% bootstrap support, respectively. The phylogenetic distance of the two-sister clusters from *C. arabica*, *C. canephora* and *C. eugenioides* confirmed they belong to a different species cluster, as suggested by the PCA analysis above. The separation of Ibo/Quirimba/*C. zanguebariae* versus the *C. racemosa* cluster was also clear; however, the current data does not allow us to assess whether this separation can be classified as a true speciation event rather than the formation of two distinct ecotypes.

## Discussion

In this work, we aimed to determine the most likely taxonomical classification of coffee samples from Ibo and Quirimba islands, which historically have been routinely confused and identified as either *C. zanguebariae* and/or *C. racemosa*. To achieve a satisfactory result, we combined two separate techniques. The first was a genome-wide analysis of SNP variants on coding genes, thus conserved across orthologs of different *Coffea* species. This produced what is to our knowledge the first large-scale sequencing and WGS characterization of Ibo coffee. The WGS analysis indicated a very narrow genetic variability within the specimens sampled across both islands and the lack of an evident genetic separation. Also, the Ibo and Quirimba samples were shown to be genetically distinct from other accession obtained from the southern districts of Mozambique. Still, the genetic distance across all Mozambican samples is much lower when compared with that of *C. canephora* and *C. eugenioides*, representing two other *Coffea* species with a diploid chromosomal asset. The second approach was a classic phylogenetic inference, with the inclusion of *bona fide* data from Davis *et al*. (2021) to allow us to compare our results with the only previous genetic characterization of Mozambican coffee reported in scientific literature. The phylogenetic tree allowed us to clearly identify *C. zanguebariae* as the taxonomical reference for both Ibo and Quirimba samples, whilst *C. racemosa* is the relative counterpart that associates to samples from the southern districts of Mozambique. Although this analysis is restricted to only four loci, the nodes are robust enough to exclude any alternative hypothesis. This work indicates that *C. racemosa* and *C. zanguebariae* are similar, but with enough genetic differentiation to be considered different species or well-defined ecotypes of the same species. Based on morphological studies, *C. zanguebariae* samples taken from Ibo and Quirimba islands can be divided into two separate variants. Interestingly, one of the two variants exhibits morphological traits of the fruits similar to those of *C. racemosa* and this may have contributed to the confusion in the characterization of the two species. The current analysis however does not indicate a genetic segregation of these two phenotypic variants, which can therefore be considered as cultivars within the species *C. zanguebariae*. This assumption could be validated in the future by crossing the A and B variants and analyzing their progenies. The availability of a reference genome for Ibo/Quirimba accessions, coupled with a larger WGS panel (A and B variants, wild and farmed specimens, additional geographical origins), may provide statistical power via GWAS and structural information to determine the contribution locus at the genome level.

This study will provide a background for further understanding of coffee from Mozambique and neighbouring regions (Tanzania) which is unique in the world of coffee farming because it relies on species different from *C. arabica, C. canephora* or *C. liberica,* which are all exploited commercially and have been crossed and selected for centuries, with little remaining potential for breeding. Having a deeper knowledge of the less studied coffee species will be of invaluable worth in the near future, as coffee cultivation suffers from the effects of climate change and new genetic material is sorely needed to introduce desirable characteristics like adaptation to higher temperatures and/or lower altitudes. It is notable that both *C. zanguebariae* and *C. racemosa* possess these characteristics, making it possible to hypothesize a future use in coffee breeding. Finally, it should not be forgotten that increasing the knowledge about these species is the essential prerequisite for modernizing the traditional agronomic methods on Ibo and Quirimba, thus improving the livelihood of local coffee farmers and safeguarding the future of this unique cultivation.

## Supporting Information

The following additional information is available in the online version of this article –

Supplementary Figure 1. Plot of PC1 and PC2, considering only samples from Ibo and Quirimba islands. Samples are divided according to the identified phenotypic variant, labelled variant A and variant B.

Supplementary Table 1. Morphological description of Variants A and B of Ibo coffee accessions sampled for the present study.

Supplementary Table 2. Identification and localization of the Ibo coffee accessions sampled for the present study.

Supplementary Table 3. Sequencing yield for each sample. Yield is reported as million reads per sample (raw Illumina reads are 150bp long).

Supplementary Code 1: ‘countHitsCategory.pl’. This Perl script was used to parse blastn results in order to detect possible contamination.

Supplementary Code 2: ‘getStatsContamination.pl’. This Perl script was used to parse blastn results in order to detect possible contamination.

plae004_suppl_Supplementary_Tables_S1-S3_Figures_S1

## Data Availability

The data underlying this article are available in the NCBI BioProject Database at https://www.ncbi.nlm.nih.gov/bioproject/, under the accession PRJNA1068845. The custom Perl scripts used to parse blastn results are provided in the Supporting Information.
