# Supplementary material for: Mozambican Coffea accessions from Ibo and Quirimba Islands: identification and geographical distribution
Source: AoB Plants. 2024 Feb 6;16(2):plae004. doi: 10.1093/aobpla/plae004 (PMC10880890; doi:10.1093/aobpla/plae004)
Supplement: plae004_suppl_Supplementary_Tables_S1-S3_Figures_S1 [file plae004_suppl_supplementary_tables_s1-s3_figures_s1.pdf]

Supplementary Figure 1: Plot of PC1 and PC2, considering only samples from Ibo and Quirimba islands. Samples are divided according to the identified phenotypic variant, labelled variant A and variant B.

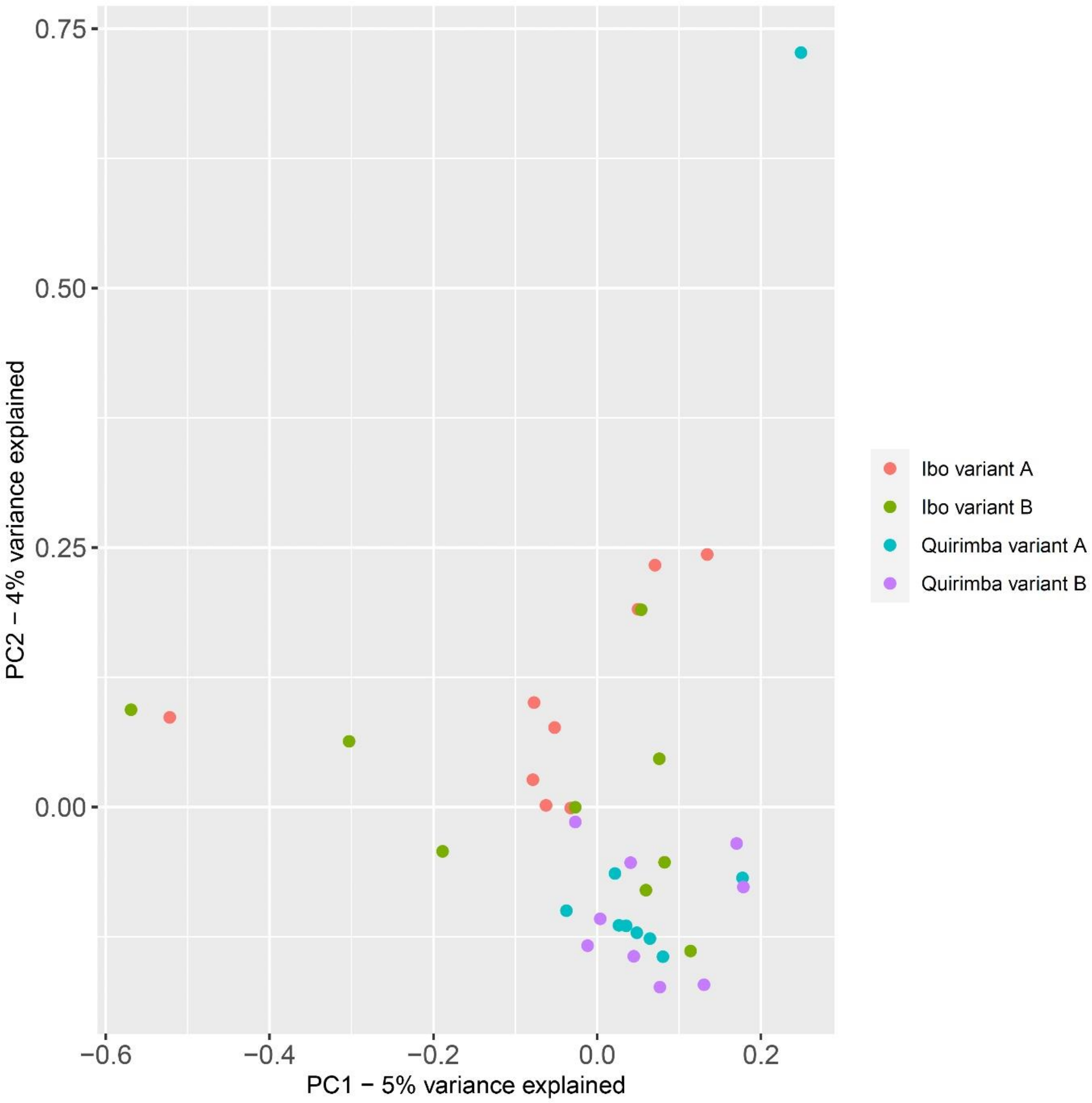

Supplementary Table 1: morphological description of Variants A and B of Ibo coffee accessions sampled for the present study.

**Character**	**Variant A**	**Variant B**
Habitus	Treelet to small tree, 3–5m tall	Treelet to small tree, 3–5.5m tall
Ramus	Scattered branching and fruiting at the ends of the branches	Dense branching and fruiting along the length of the branches
Flower (corolla)	8 petals (in a very few cases 7 or 9)	8 petals (in a very few cases 7 or 9)
Fruit size (average on 100 units)	12.75 mm (length)<br>8.4 mm (width)<br>6.75 mm (depth)	9.25 mm (length)<br>7.85 mm (width)<br>6.55mm (depth)
Fruit shape	narrow and long, elliptical, smooth and hairless	spherical, short, smooth and hairless
Fruit color (full maturation)	Ripe fruit are brown, becoming a darker shade of brown as the fruit dries	Ripe fruit are brown, becoming a darker shade of brown as the fruit dries
Fruit color (unripe)	Unripe fruits are green with remarkable longitudinal veins	Unripe fruits are green with no remarkable longitudinal veins
Fruit weight (average on 100 units)	57.05 g	44.1g
Seed shape	flat, pointed and long	round and small
Seed size (on average of 100 gr)	11.1mm (length)<br>5.4 mm (width)<br>3.7 mm (depth)	8.5mm (length)<br>5.8 mm (width)<br>4.2 mm (depth)
Seed weight (average on 100 gr)	8.5 g	7.8 g

Supplementary Table 2: identification and localization of the Ibo coffee accessions sampled for the present study.

Cod	Taxonomia	Province	District	Coordinate	
				Latitude	Longitude
**1**	*Coffea racemosa*	Inhambane	Maxixi	-23,873014	35,338042
**3**	*Coffea racemosa*	Inhambane	Inharrime	-24,460833	34,163889
**9**	*Coffea racemosa*	Inhambane	Zavala	-24,718388	34,742919
**10**	*Coffea racemosa*	Inhambane	Zavala	-24,716259	34,746567
**342**	Variante B	C. Delgado	Ibo	-12,335846	40,5900152
**401**	Variante B	C. Delgado	Ibo	-12,3506002	40,5977048
**402**	Variante B	C. Delgado	Ibo	-12,350553	40,5978889
**403**	Variante B	C. Delgado	Ibo	-12,3503354	40,5974873
**405**	Variante A	C. Delgado	Ibo	-12,3496795	40,6004028
**422**	Variante A	C. Delgado	Ibo	-12,34971	40,6004828
**423**	Variante A	C. Delgado	Ibo	-12,3498088	40,6000654
**425**	Variante B	C. Delgado	Ibo	-12,3491583	40,6001285
**426**	Variante B	C. Delgado	Ibo	-12,3497945	40,6000456
**428**	Variante A	C. Delgado	Ibo	-12,3485911	40,5999454
**429**	Variante B	C. Delgado	Ibo	-12,349148	40,6000836
**432**	Variante B	C. Delgado	Ibo	-12,3356714	40,5894987
**434**	Variante B	C. Delgado	Ibo	-12,3356956	40,5894273
**437**	Variante A	C. Delgado	Ibo	-12,3345345	40,5879942
**471**	Variante A	C. Delgado	Ibo	-12,3424195	40,5927449
**473**	Variante A	C. Delgado	Ibo	-12,3424359	40,5927132
**477**	Variante A	C. Delgado	Ibo	-12,3506162	40,5977994
**480**	Variante A	C. Delgado	Ibo	-12,3506296	40,5978833
**2**	Variante A	C. Delgado	Quirimba	-12,417634	40,6019754
**4**	Variante A	C. Delgado	Quirimba	-12,4185351	40,6019355
**5**	Variante B	C. Delgado	Quirimba	-12,4181187	40,6019944
**13**	Variante B	C. Delgado	Quirimba	-12,4154844	40,6016225
**341**	Variante B	C. Delgado	Quirimba	-12,4137856	40,6075594
**348**	Variante A	C. Delgado	Quirimba	-12,4130436	40,607921
**351**	Variante B	C. Delgado	Quirimba	-12,4135879	40,6056151
**352**	Variante A	C. Delgado	Quirimba	-12,4138713	40,6055896
**353**	Variante B	C. Delgado	Quirimba	-12,4137751	40,6052322
**356**	Variante A	C. Delgado	Quirimba	-12,4135979	40,6055854
**359**	Variante B	C. Delgado	Quirimba	-12,4394437	40,6072805
**410**	Variante A	C. Delgado	Quirimba	-12,4394693	40,6085133
**411**	Variante B	C. Delgado	Quirimba	-12,4394781	40,6084353
**412**	Variante A	C. Delgado	Quirimba	-12,4394444	40,6083268
**413**	Variante B	C. Delgado	Quirimba	-12,4136897	40,6074412
**438**	Variante B	C. Delgado	Quirimba	-12,4185867	40,6024004
**440**	Variante A	C. Delgado	Quirimba	-12,4187895	40,6023565
**478**	Variante A	C. Delgado	Quirimba	-12,4126808	40,6073587

Supplementary Table 3: sequencing yield for each sample. Yield is reported as million reads per sample (raw Illumina reads are 150bp long).

**Sample Name**	**Million Reads**
1-Maxixi	159.53
3-Inharrime	145.77
9-Zavala	156.98
10-Zavala	176.93
342-Ibo	163.64
401-Ibo	137.21
402-Ibo	171.38
403-Ibo	155.31
405-Ibo-A02	250.99
422-Ibo-B02	273.24
423-Ibo-C02	233.80
425-Ibo-D02	146.66
426-Ibo-E02	286.08
428-Ibo-F02	183.21
429-Ibo-G02	205.92
432-Ibo-H02	210.46
434-Ibo-A03	291.16
437-Ibo-B03	235.81
471-Ibo-C03	251.59
473-Ibo-D03	218.50
477-Ibo-E03	216.40
480-Ibo-F03	240.38
2-Quirimba-G03	236.11
4-Quirimba-H03	231.00
5-Quirimba-A04	215.47
13-Quirimba-B04	267.96
341-Quirimba-C04	159.35
348-Quirimba-D04	192.11
351-Quirimba-E04	176.35
352-Quirimba-F04	152.20
353-Quirimba-G04	310.09
356-Quirimba-H04	139.58
359-Quirimba-A05	232.78
410-Quirimba-B05	303.38
411-Quirimba-C05	181.20
412-Quirimba-D05	217.89
413-Quirimba-E05	153.63
438-Quirimba-F05	199.82
440-Quirimba-G05	240.73

Supplementary Code 1: "countHitsCategory.pl". This Perl script was used to parse blastn results in order to detect possible contamination.

```
#!/usr/bin/env perl
###################################################################################
# Description: takes a set of words matching different features and a blast output #
#              reports for each category the number of hits matching given words   #
###################################################################################

use warnings;
use strict;
use Getopt::Long;
use Pod::Usage;
use Switch;
my $categories;
my $blast;
my $help = 0;

GetOptions(
	'categories=s' => \$categories,
	'blast=s' => \$blast,
	'help|?'       => \$help
) or pod2usage(2);
pod2usage(1) if $help;
pod2usage(1) if (!defined($categories) or !defined($blast));

my %categories;
open(CATEGORIES, $categories) or die("Could not open input file $categories\n");
while (my $line = <CATEGORIES>) {
	chomp $line;
	if ($line =~ /Category/) { next; }
	if ($line =~ /(\S+)\t(\S+)/) {
		$categories{$1}{hits} = 0;
		$categories{$1}{words} = $2;
	}
	else {
		die($line." does not match given syntax for file of categories $categories\n");
	}
}
close CATEGORIES;

open(BLAST, $blast) or die("Could not open input file $blast\n");
while (my $line = <BLAST>) {
	chomp $line;
	if ($line =~ /<Hit_def>(.+)<\/Hit_def>/) {
		my $hit_def = $1;
		foreach my $category (keys %categories) {
			my $discovered = 0;
			foreach my $word (split(/,/,$categories{$category}{words})) {
				if ($hit_def =~ /$word/i) { $discovered = 1; }
			}
			if ($discovered) { $categories{$category}{hits}++; }
		}
	}
}
close BLAST;

foreach my $category (keys %categories) {
	print $categories{$category}{hits}."\t".$category."\n";
}

__END__


=head1 GFF Statistics

countHitsCategory.pl - Using this script

=head1 SYNOPSIS

perl countHitsCategory.pl [--categories <filename>] [--blast <filename>] [--help]

  Options:
    --categories <filename>       input file, required. Format, each category on separate lines: "category TAB comma separated list of words for current category. E.g. ribosomal	ribos,rRNA. See also /iga/scripts/scalabrin/parsers/specific_categories.txt
    --blast <filename>            output of blastn in xml format, required
    --help                        print this help message

output on standard output (redirect, if necessary)
```

Supplementary Code 2: “getStatsContamination.pl”. This Perl script was used to parse blastn results in order to detect possible contamination.

```
#!/usr/bin/env perl
###############################################################
# Description: takes a two columns file and returns percentages #
###############################################################

use warnings;
use strict;
use Getopt::Long;
use Pod::Usage;
use Switch;
my $input;
my $query_number = 50000;
my $help    = 0;

GetOptions(
        'input=s' => \$input,
        'query_number=i' => \$query_number,
        'help|?'       => \$help
) or pod2usage(2);
pod2usage(1) if $help;
pod2usage(1) if (!defined($input));

my $sum=0;
open(INPUT, $input) or die("Could not open input file $input\n");
while (my $line = <INPUT>) {
        chomp $line;
        if ($line =~ /\s*(\d+)\s+(\S+)/) {
                $sum += $1;
        }
        else {
                die($line." does not match\n");
        }
}
close INPUT;

open(INPUT, $input) or die("Could not open input file $input\n");
print "Category\tCount\tRelative ratio\tAbsolute ratio\n";
while (my $line = <INPUT>) {
        chomp $line;
        if ($line =~ /\s*(\d+)\s+(\S+)/) {
                my $count = $1;
                my $genus = $2;

                # control ratio
                my $ratio = 0;
                if ( $sum != 0 ) {
                        $ratio = int($count * 10000 / $sum) / 100;
                }

                my $absolute_ratio = int($count * 10000 / $query_number) / 100;
                print $genus."\t".$count."\t".$ratio."\%\t".$absolute_ratio."\%\n";
        }
        else {
                die($line." does not match\n");
        }
}
close INPUT;

__END__


=head1 GFF Statistics

getStats.pl - Using this script

=head1 SYNOPSIS

perl getStats.pl [--input <filename>] [--query_number <integer>] [--help]

  Options:
    --input <filename>            input file, required
    --query_number <integer>      number of input queries to compute the absolute percentage of hits
    --help                        print this help message

output on standard output (redirect, if necessary)
```
